# Diagnostic Limitations of Applying a Human Portable Blood Glucose Meter in the Detection of Hypoglycemia in Pregnant Ewes

**DOI:** 10.3390/vetsci12010047

**Published:** 2025-01-11

**Authors:** José Lucas Xavier Lopes, Raquel Fraga e Silva Raimondo, Luiza Rodegheri Jacondino, Beatriz Riet Correa, Clara Satsuki Mori, Álan Gomes Pöppl

**Affiliations:** 1 Programa de Pós-Graduação em Ciências Veterinárias, Universidade Federal do Rio Grande do Sul (UFRGS), Porto Alegre 91540-000, Brazil; 2 Núcleo RuminAção, Ensino, Pesquisa e Extensão em Ruminantes, Faculdade de Veterinária, Universidade Federal do Rio Grande do Sul (UFRGS), Porto Alegre 91540-000, Brazil; 3 Departamento de Medicina Animal, Faculdade de Veterinária, Universidade Federal do Rio Grande do Sul (UFRGS), Porto Alegre 91540-000, Brazil; 4 Programa de Pós-Graduação em Clínica Veterinária, Faculdade de Medicina Veterinária e Zootecnia, Universidade de São Paulo (USP), São Paulo 05508-270, Brazil; 5 Programa de Pós-Graduação em Zootecnia, Universidade Federal do Rio Grande do Sul (UFRGS), Porto Alegre 91540-000, Brazil

**Keywords:** sheep, blood glucose, Accu-Check Performa, ISO 15197:2013

## Abstract

Pregnant ewes are susceptible to hypoglycemia and ketosis; therefore, monitoring glycemic status is extremely important. Portable blood glucose meters (PBGMs) can assist in quickly and conveniently identifying glycemic disturbances in this species, provided that they meet the criteria of analytical accuracy. This study evaluated the performance of a human PBGM (Accu-Chek Performa^®^, Roche Diagnostics, Basel, Switzerland) in the glycemic evaluation of 34 pregnant ewes at days 90 and 120 of pregnancy in comparison with the results of glycemia determination by a reference method (RM). The device showed a high positive correlation (r = 0.71, 95%CI = 0.57–0.82, *p* < 0.0001) with the RM; however, 96.6% of the PBGM results (58.5 ± 9.82 mg/dL) were higher (*p* < 0.0001) than those obtained in the laboratory (48.6 ± 9.31 mg/dL). The PBGM tested was considered analytically inaccurate according to ISO 15197:2013, which states that when glucose levels are below 100 mg/dL, 95% of the measurements should deviate by no more than 15 mg/dL from the RM value, and 1/3 of the PBGM results were above this limit. Hypoglycemia (<50 mg/dL) was documented in 60.29% of samples tested on with the RM, but only 17.64% of results were below 50 mg/dL using the PBGM. Due to these limitations, Accu-Check Performa^®^ results should be interpreted cautiously in pregnant sheep suspected of hypoglycemia.

## Simple Summary

Hypoglycemia (low blood sugar) and ketosis are common metabolic disorders in pregnant ewes, highlighting the importance of accurate blood glucose monitoring. The portable blood glucose meter (PBGM) is a fast and practical tool for detecting these conditions, but its reliability must be confirmed. This study assessed the accuracy of a human PBGM (Accu-Chek Performa^®^, Roche Diagnostics, Basel, Switzerland) in 34 pregnant ewes at 90 and 120 days of gestation. While the PBGM results showed a moderate to strong correlation with laboratory measurements, it consistently overestimated blood glucose levels, leading to the unreliable detection of hypoglycemia. These results indicate that, despite their convenience, PBGMs should be used with caution for diagnosing hypoglycemia in pregnant ewes.

## 1. Introduction

Sheep had higher glucose demands in the gestational third (final) period, as well as due to twin pregnancy, in response to accelerated fetal growth and limited ruminal capacity. Mammary gland development also accounts for increased glucose requirements. In some cases, glucose demands are not fully supplied to facilitate normoglycemia (50–80 mg/dL), resulting in hypoglycemia, increased lipolysis, and eventual ketosis [1]. Peripartum glycemia monitoring helps identify metabolic disorders and can be considered a useful indicator of fetal viability [2].

Portable blood glucose meters (PBGMs) are often applied for glycemia determination in clinical routines for small animals [3]. In ruminant medicine, PBGMs can be a useful tool for glucose homeostasis monitoring due to the quick results and cost reductions they allow for compared with the method of shipping samples to a laboratory [4]. There is no specific PBGM for use in ruminants in the Brazilian market; however, several devices developed for human or veterinary use are available and validation studies for their accurate use in ruminants are warranted. Therefore, this study aimed to assess the analytical precision of the PBGM device Accu-Chek Performa^®^, developed for humans, to assess glycemia in pregnant sheep.

## 2. Materials and Methods

### 2.1. Animals and Procedures

Thirty-four pregnant sheep with no defined breed and a mean body condition score of 3 (1–5) kept in a pasture system were included in the study by convenience sampling. The pasture comprised native Pampa grassland with ryegrass overseeding, a common condition in the Rio Grande do Sul state, Southern Brazil, where the study was performing. Blood samples were drawn at the gestational ages of 90 and 120 days by jugular venipuncture with a Vacutainer system with a 25 × 0.8 mm needle in two vacuum plastic tubes, one with sodium fluoride/EDTA and the other with clot activator and no anticoagulant. All samples were drawn in the morning at around 9–10 a.m. by experienced researchers, and no problems regarding blood obtention or severe stress among the ewes were registered.

Glycemia was determined immediately from the tube without anticoagulant after sampling with a human PBGM (Accu-Chek Performa^®^, Roche Diagnostics, Basel, Switzerland), as preconized by Raimondo et al. [5]. According to the manufacturers, the minimum blood volume required by the device is 0.6 μL, and the blood glucose detection range is 10 to 600 mg/dL. The device operates without hematocrit interference within the 10–65% range, and an enzymatic reaction of glucose dehydrogenase is used in the test strips.

After clot retraction, samples were centrifugated at 700× *g* for 15 min (Centrifuge 80-2b, Daiki^®^, Changshu, China) and the resultant serum was separated in microtube aliquots and kept frozen at −20 °C until analysis. For the analytical precision evaluation of the PBGM, the glycemia results obtained with the portable device were compared with those from a reference method (RM—Glucose Kit, Labtest, Lagoa Santa, Brazil) using an automatic biochemical analyzer (Labmax 240, Tokyo Boeki, Tokyo, Japan) at the Biochemistry Laboratory (Departamento de Clínica Médica, FMVZ, USP).

### 2.2. Statistical Analyses

The analytical accuracy was evaluated against the ISO 15197:2013 [6] requirements for human PBGMs [6]. Two conditions should be met for a PBGM to be considered accurate: (1) for glycemia below 100 mg/dL, 95% of its results must not differ by more than 15 mg/dL from the RM result, and (2) for glycemia equal or greater than 100 mg/dL, 95% of its results must not differ by more than 15% from the RM result.

The GraphPad Prism 6 software package (GraphPad Software Inc., San Diego, CA, USA) was used for statistical analyses. The Shapiro–Wilk test was applied to assess data normality. The PBGM and RM results were compared using the paired *t*-test for accuracy assessment, and the differences between values are represented by the Bland–Altman plot [7]. The paired *t*-test was also applied to compare the glycemic data from day 90 with those from day 120. Glycemic data from twin-pregnant ewes were compared with mono-pregnant ewes by the *t*-test. Results were expressed as mean ± standard deviation. Also, the Pearson coefficient was determined to assess the correlation between the results. The interpretation of the correlation coefficient values was as follows: 0.9–1, very high; 0.7–0.89, high; 0.5–0.69, moderate; 0.3–0.49, low; and 0–0.29, minimal correlation [8,9,10]. Differences were considered significant at a *p*-value < 0.05.

## 3. Results

### 3.1. Glycemic Range Categorization According to Each Measurement Method

According to the PBGM or RM reads, patients were classified as hypoglycemic (<50 mg/dL), normoglycemic (50–80 mg/dL), or hyperglycemic (>80 mg/dL) in accordance with Kaneko et al. [11]. Table 1 shows the number and percentage of patients considered to be in each glycemic range according to the glycemia determination method. Most samples were in the hypoglycemic range measured by the RM (60.29%); however, only 17.64% of the PBGM reads indicated hypoglycemia. Analyzing data from the different pregnancy times studied, hypoglycemia was documented in 3/34 (8.82%) of the samples measured by the PBGM against 16/34 (47.06%) of the samples measured by the RM on the 90-day. On day 120, samples measured by the PBGM showed hypoglycemia in 9/34 (26.47%) ewes, against 25/34 (73.52%) in samples measured by the RM.

Reproducibility analysis of the results by each method in the pregnancy moments evaluated showed no difference (*p* = 0.1095) in mean glycemia on day 90 measured by the PBGM (60.92 ± 9.2 mg/dL) compared with that on day 120 (57.2 ± 10.2 mg/dL); however, results obtained by the RM showed a statistically significant difference (*p* < 0.001) between day 90 (51.28 ± 7.9 mg/dL) and day 120 (43.9 ± 9.1 mg/dL).

### 3.2. Inaccuracy of the PBGM Results Compared to the Reference Method

**Figure 1:**
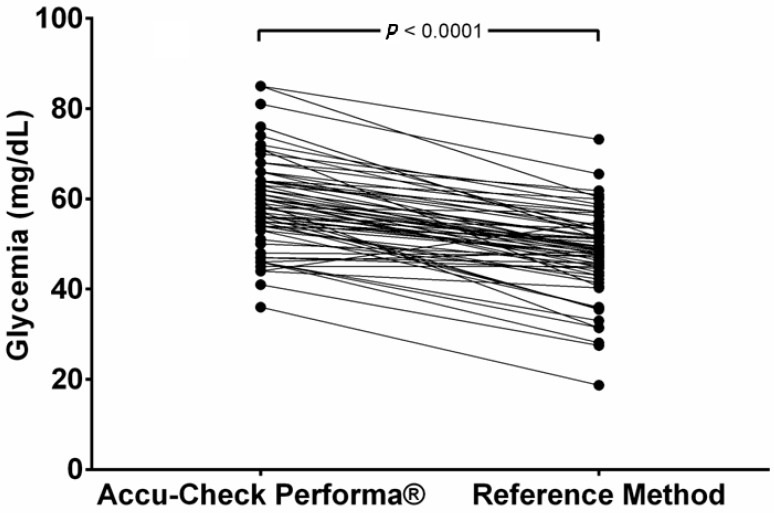
Paired comparison of glycemia results obtained using a portable blood glucose meter (PBGM) designed for human use (Accu-Check Performa^®^) or a laboratory reference method (RM) in pregnant ewes at days 90 and 120 of pregnancy.

Only two samples (2.9%) showed PBGM values below those obtain by the RM, and one (1.5%) showed the same results in both methods. The other 65 samples (96.6%) had higher values from the PBGM. The mean glucose values read by the PBGM (58.5 ± 9.82 mg/dL) were significantly higher (*p* < 0.0001) than the results obtained by the RM (mean = 48.6 ± 9.31 mg/dL) and are represented in Figure 1. This distortion means that the PBGM suggested that 4.41% of the samples indicated hyperglycemia (>80 mg/dL), while no hyperglycemic values were documented using the RM.

**Figure 2:**
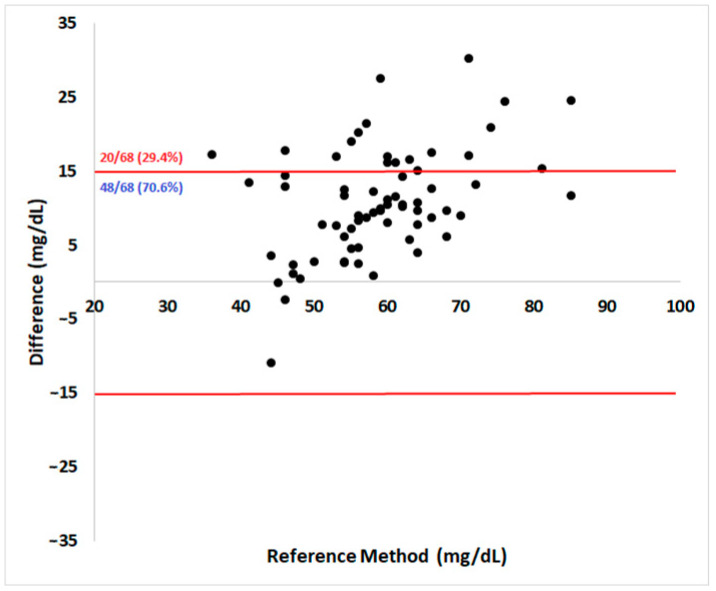
Bland–Altman plot of the portable blood glucose meter (PBGM) Accu-Check Performa^®^’s measurements evaluated in pregnant ewes at days 90 and 120 of pregnancy. The x-axis shows glycemia determined by the reference method and the y-axis shows the corresponding absolute difference between the PBGM and the RM results. The limits defined by ISO 15197:2013 for analytical accuracy are represented as red lines. A PBGM should have no more than 5% of its reads outside the established limit to be considered accurate.

Regarding analytical accuracy according to the ISO 15197:2013 guidelines, 70.6% of the results were within the acceptable ± 15 mg/dL absolute error range for samples below 100 mg/dL (Figure 2). The Pearson coefficient showed a high correlation (r = 0.71) between methods (95% confidence interval = 0.57–0.82, *p* < 0.0001).

### 3.3. Differences According to Twin Pregnancy Occurrence

Twin pregnancy was documented in 7 out of 34 pregnant ewes (20.59%). The mean glycemia determined by the RM in twin-pregnant ewes on day 90 was 45.8 ± 6.8 mg/dL, while mono-pregnant ewes showed a mean glycemia value of 52.6 ± 6.2 mg/dL (*p* = 0.8691). Notwithstanding, when glycemia results determined by the PGBM were compared, twin-pregnant ewes showed smaller (*p* = 0.0132) mean glycemia values (53.3 ± 5.8 mg/dL) compared with mono-pregnant ewes (62.7 ± 9.1 mg/dL). The same comparisons were provided for the analysis on day 120. The mean glycemia value determined by the RM in twin-pregnant ewes (35.4 ± 10.3 mg/dL) was significantly smaller (*p* = 0.0036) than that in mono-pregnant ewes (46.2 ± 7.4 mg/dL). In contrast, data obtained by the PBGM did not show statistical differences (*p* = 0.1465) between mean glycemia from twin-pregnant ewes (52.1 ± 9.7 mg/dL) compared with the mono-pregnant ones (58.4 ± 10.1 mg/dL).

## 4. Discussion

Despite the moderate to high correlation between evaluated methods [8,9,10], the higher glycemia results read by the PBGM in the majority of tested samples represent a potential risk for hypoglycemia misdiagnosis in ewes since only two-thirds of the samples were within the acceptable absolute error of 15 mg/dL preconized by the ISO 15197:2013 for samples below 100 mg/dL. To achieve ISO validation for analytic precision, a PBGM should have more than 95% of reads within acceptable limits [6]. Notwithstanding, it is important to emphasize that ISO recommendations for PBGM validation might not be completely valid for glycemia evaluation in ruminants. A <15 mg/dL bias in animals in which normal glycemia ranges between 50 and 80 mg/dL [11] may be enough to cause diagnostic errors, as evidenced in the present study, since the 41 samples considered hypoglycemic by the RM, amounting to about 70%, were read as normoglycemic by the PBGM.

According to the PBGM manufacturer, this device operates without hematocrit interference in humans with packet cell volumes between 10 and 65%. The fact that the ewe’s hematocrit was not evaluated in this case could be considered a limitation of the present study. However, ewes’ hematocrit often ranges between 28 and 33% during pregnancy, which is within the reference range for the species, and no effect of pregnancy on hematocrit leading to hemodilution has been reported in ewes [12,13]. The lower the number of erythrocytes in a whole-blood sample, the greater the volume of plasma that penetrates the test strip reagent layer, resulting in inaccurate results. In this way, hemodilution produces higher glycemic values, while hemoconcentration leads to lower values in PBGMs [14].

Another critical point that could have interfered with the glycemic results obtained by the RM was the time for sample centrifugation. Quick blood sample centrifugation after sampling is crucial to avoid time interference in the glycemia results obtained by the reference method. Despite the inhibitory effect of sodium fluoride within EDTA tubes on glycolysis, the process is not completely interrupted. Without glycolysis inhibition by the anticoagulant, glycemia may reduce by 5–7% per hour within the sample while waiting to be centrifugated due to erythrocyte metabolism [15]. In this way, blood sampling in sodium fluoride EDTA tubes and prompt centrifugation after clot retraction could potentially minimize bias in the glycemic results obtained by an RM.

An absolute variation greater than 10% was documented in 33% of the samples in a study evaluating PBGM precision in ruminants, including sheep. The same survey showed a variation greater than 20% in 10% of the ewes’ samples [16]. Despite these results not fulfilling ISO standards for analytical accuracy, the PBGM was considered proper for use in the field. Moreover, total blood samples may provide different results than plasma [17]; however, the practicality of PBGM use in the stockyard precludes total blood use, and comparisons with reference methods are often made in plasma samples.

It is important to mention that the Accu-Check Performa^®^ device is considered reliable for human use due to its adherence to the accuracy criteria of ISO 15197:2013 [18]; notwithstanding, the PBGM validity documented for a given species (i.e., human, dog, cat) does not equate to reliable performance for any species [3], warranting studies like this one to be conducted before applying PBGM developed for use in other species in ruminant medicine.

Finally, this study aimed to test glycemia on pregnancy days 90 and 120 because these periods are near the end of the second-third and middle of the final-third pregnancy phase, respectively. The final pregnancy period is marked by 30–40% consumption of the glucose produced by the fetoplacental unit when the fetus develops up to 80% of its birth weight [19]. A negative energy balance often results from the ewe’s increased energy needs, associated with compressed ruminal space, leading to reduced dry matter consumption [1,2,19]. Twin pregnancy could further unbalance an ewe’s energy status [4].

The use of the tested PBGM in this study was not just associated with hypoglycemia underestimation in the herd but also negatively impacted the ability to distinguish hypoglycemia trends between pregnancy days 90 and 120, and underestimated hypoglycemia in twin pregnancies. Moreover, the reliability of glycemia as a marker of fetal viability in the peripartum [2] would be negatively impacted, despite the present study not evaluating peripartum glycemia. These findings further emphasize the potential bias of adopting PBGM designed for other species, particularly in the glycemic monitoring of pregnant ewes.

## 5. Conclusions

In this way, a desirable PBGM should provide quick and accurate results to provide a correct diagnosis and facilitate the metabolic monitoring of common conditions in pregnant ewes, such as hypoglycemia and pregnancy ketosis. The minimal blood sample volume required to obtain sheep’s glycemia using PBGMs is also an advantage of these devices. However, caution should be applied when interpreting glycemic results in ewes suspected of being hypoglycemic, since the tested device may perform poorly for hypoglycemia detection in this species. Reflecting the fact that the ISO requirements were not fully applicable to ruminant glycemia validation, the inconsistency herein reported supports the assumptions that PBGM Accu-Check Performa^®^ was not analytically accurate for hypoglycemia detection in pregnant ewes and that it may result in serious misdiagnosis. Therefore, the usefulness of this PBGM to detect hypoglycemia in pregnant ewes should be discouraged.

## Figures and Tables

**Table 1:** The total number and percentual of samples classified as hypoglycemic (values < 50 mg/dL), normoglycemic (values between 50 and 80 mg/dL), and hyperglycemic (values > 80 mg/dL) in pregnant ewes at days 90 and 120 of pregnancy according to the glycemia determination method.

	Hypoglycemia (<50 mg/dL)	Normoglycemia (50–80 mg/dL)	Hyperglycemia (>80 mg/dL)
**Accu-Check Performa^®^**	12 (17.64%)	53 (77.94%)	3 (4.41%)
**Reference Method**	41 (60.29%)	27 (39.71%)	0 (0%)

## Data Availability

Materials and data sheets are available upon request to interested researchers.
